# Pan-American Medical Congress in Columbia

**Published:** 1892-02

**Authors:** Charles A. L. Reed

**Affiliations:** Secretary General; Cincinnati


CHARLES A. L. REED,

Secretary General.

Cincinnati, January 15, 1892.

### The Pan-American Medical Congress in the United States of Columbia.

Pursuant to nominations by Dr. Pedro M. Ibañez, of Bogota, member of the International Executive Committee for the United States of Columbia, the following organization of the Pan American Medical Congress has been effected in that country: Vice-President, Dr. Pio Rengifo, New York; Secretaries of Sections: General Medicine, Dr. Ignacio Guttierrez Ponce, Paris; General Surgery, Dr. Rafael Rocha Castilla, Bogota; Military Medicine and Surgery, Dr. Abraham Aparicio, Bogota; Obsteterics, Dr. Joaquin Maldanado, Bogota; Gynecology and Abdominal Surgery, Dr. Jose M. Buendia, Bogota; Therapeutics, Dr. Manuel Plata Azuero, Guaduas; Anatomy, Dr. Joan D. Herrara, Bogota; Physiology, Dr. Antonio Bargas Vega, Bogota; Pathology, Dr. Nicolas Osorio, Bogota; Diseases of Children, Dr. Ant. Gomez Calvo, Bogota; Laryngology and Rhinology, Dr. Luis Fonnegra, Bogota; Otology, Dr. Carlos Esquerra, Bogota; Dermatology, Dr. Daniel E. Coronado, Bogota; Orithopædics, Dr. Juan E. Manrique, Bogota; Naval Hygiene and Quarantine, Gabriel I. Castanada, Bogota; General Hygiene and Demogrophy, ———; Mental and Nervous Diseases, Dr. Pablo Garcia Medina, Bogota; Oral and Dental Surgery, Dr. Guillermo Vargas Paredes, Bogota; Medical Pedagogics, Dr. Jorge Vargas, Bogota; Medical Jurisprudence, Dr. Leoncio Barreto, Bogota.

Auxiliary Committee (each member being the official representative of the congress in his respective city).—Dr. Nicolas Osorio, Dr. Andres Posada Arango, Dr. Jorge E. Delgado, Dr. Eugeneo de la Hoz, Dr. Domingo Cagiao, Dr. Jose Manuel Rodrigues, Dr. Paulo Emilio Villar, Dr. Felix M. Hernandez, Dr. Rafael Calvo, Dr. N. Ribòn, Dr. Nilceades Castro; Dr. Cayetano Lombane, Dr. José M. Martinez, Dr. Isaias Saavedra, Dr. Severo Torres, Dr. N. Villa, Dr. Evarista Garcia, Dr. Miguel Caicedo, Dr. Emilio Villamizar.

The following medical societies have been elected as auxiliaries of the congress, viz.: Academia Nacional de Medicina, Academia de Medicina de Medellin, Sociedad de Medicia del Cauca.

The following medical journals have been designated as official organs of the congress, viz: *Revista Medica*, Bogota; *Revista de Higene*, Bogota; *El Agricutor*, Bogota; *Boletin de Medicina del Cauca*, Cali; *Anales de la Academia de Medicina de Medellin*, Medellin.

The expressed wish of the profession of the United States of Columbia is for a date of meeting during the Columbian Exposition.

